# Supplementary material for: Mechanical cleaning of graphene using in situ electron microscopy
Source: Nat Commun. 2020 Apr 8;11:1743. doi: 10.1038/s41467-020-15255-3 (PMC7142133; doi:10.1038/s41467-020-15255-3)
Supplement: Supplementary file 1 — Supplementary Information [file 41467_2020_15255_MOESM1_ESM.pdf]

Mechanical cleaning of graphene using *in situ* electron microscopy – Supplementary Information

*Peter Schweizer[1], Christian Dolle[1], Daniela Dasler[2], Gonzalo Abellán[2,3], Frank Hauke[2], Andreas Hirsch[2], Erdmann Spiecker[1]**

[1] Institute of Micro- and Nanostructure Research and Center for Nanoanalysis and Electron Microscopy (CENEM), FAU Erlangen-Nürnberg, Cauerstr. 3, 91058, Erlangen, Germany

[2] Department of Chemistry and Pharmacy and Joint Institute of Advanced Materials and Processes (ZMP), Chair of Organic Chemistry II, FAU Erlangen-Nürnberg, Nikolaus-Fiebiger-Str. 10, 91058, Erlangen, Germany

[3] Instituto de Ciencia Molecular (ICMol), Universidad de Valencia, Carrer del Catedrátic José Beltrán Martinez, 2, 46980 Paterna, Valencia, Spain

Supplementary Figures

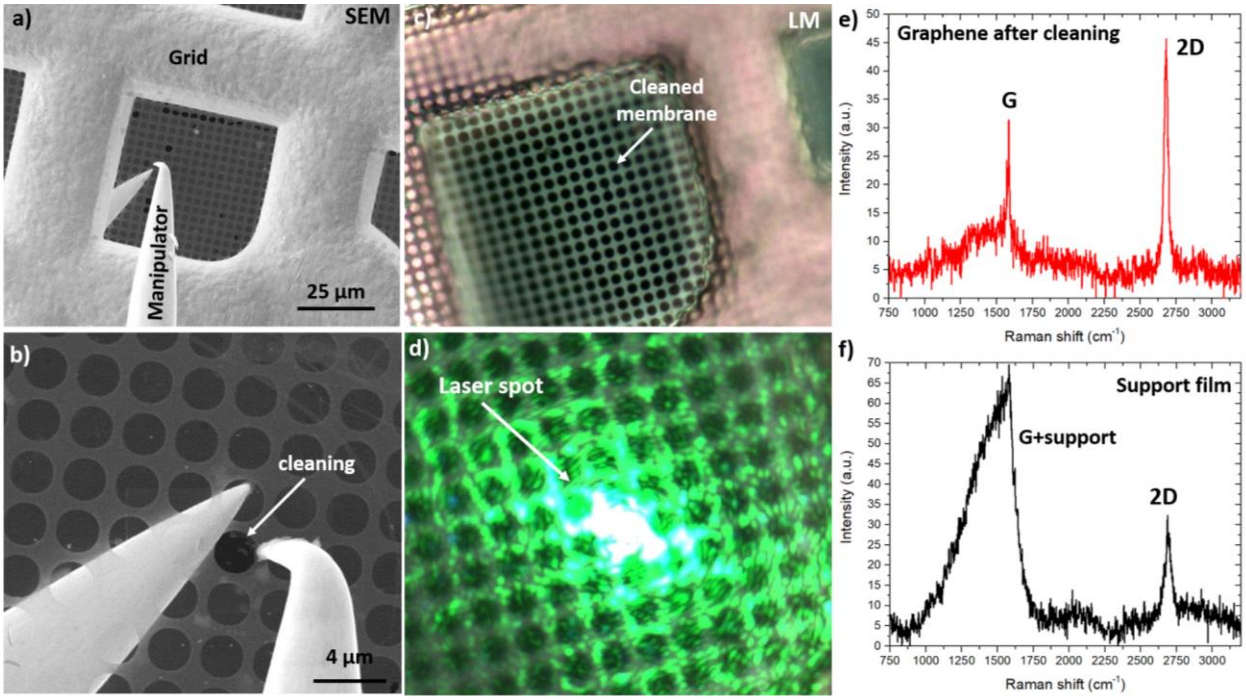

Supplementary Figure 1: Raman measurement of mechanically cleaned graphene. a) Overview image of the cleaning setup and close-up of a cleaned window (b). c) Optical microscopy image (100 x objective) of the same sample area. d) Superimposed laser spot used for Raman measurements. Even using the highest magnification, a considerable spread of the laser spot is visible. e) Raman spectrum of mechanically cleaned graphene, which does not show an increase in D-band intensity. The support film contributes intensity in a broad range from 1000 cm^{-1} to around 1600 cm^{-1} (f). The spectra were measured at 532 nm.

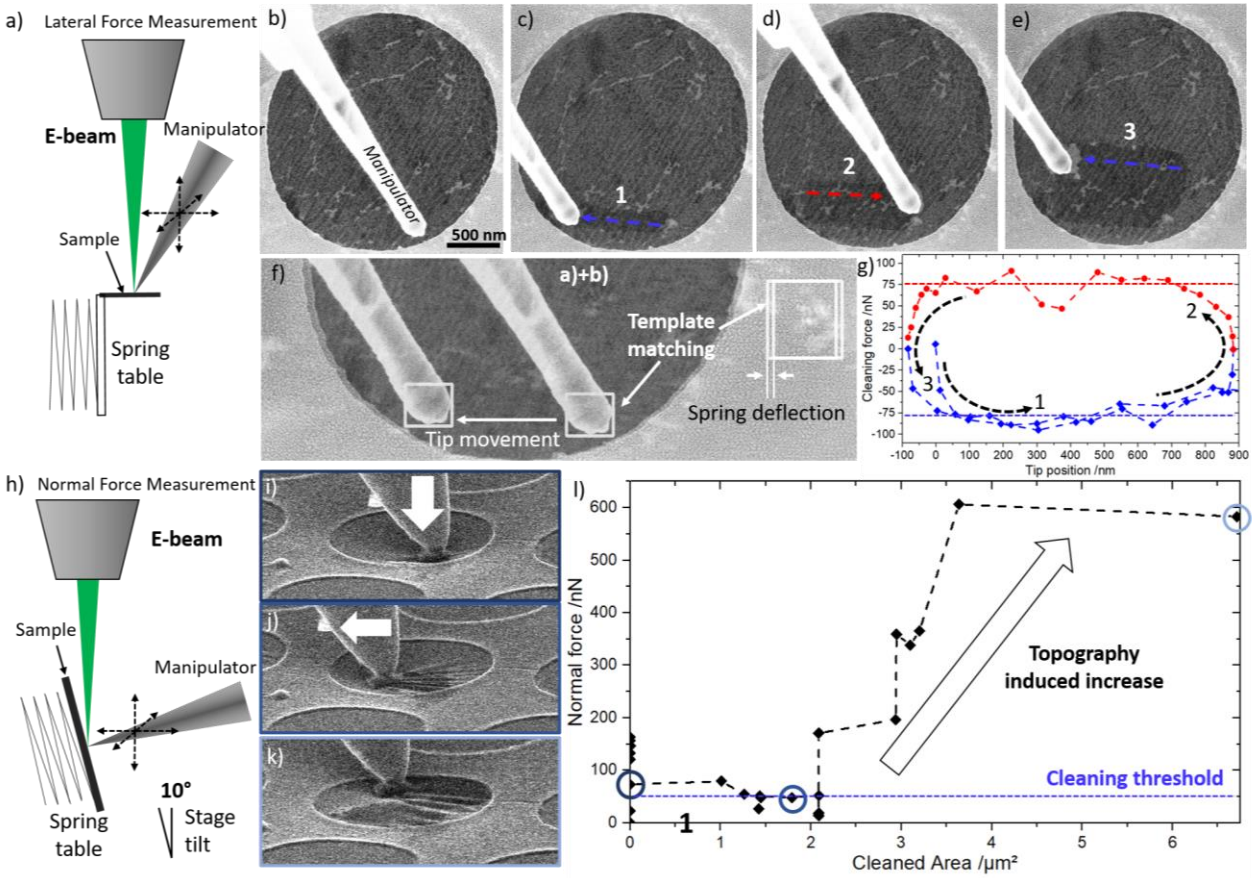

Supplementary Figure 2: Cleaning force measurement in lateral and normal direction. a) Setup for cleaning in lateral direction. The sample is placed on a spring table which deflects in one direction upon loading. During cleaning (b-e), small horizontal deflections of the sample are induced, depending on the direction of movement of the manipulator. To analyze these deflections, automated template matching is performed on the undeformed support grid, as well as the manipulator tip (f). With the known spring constant of 11 N/m, a force-displacement curve can be calculated (g). To measure normal forces, the sample normal is aligned along the direction of spring-deflection (h). In order to see the cleaning process, the microscope stage as a whole is tilted by 10°. During cleaning (i-k) the deflection in membrane normal direction can be evaluated in the same way as in the lateral direction. Evaluation these forces, a minimal normal force for cleaning of 50 nN was found (l). The increase in normal force with the cleaned area is a result of the tilted geometry, which has not been compensated for by additional lateral movements of the manipulator.

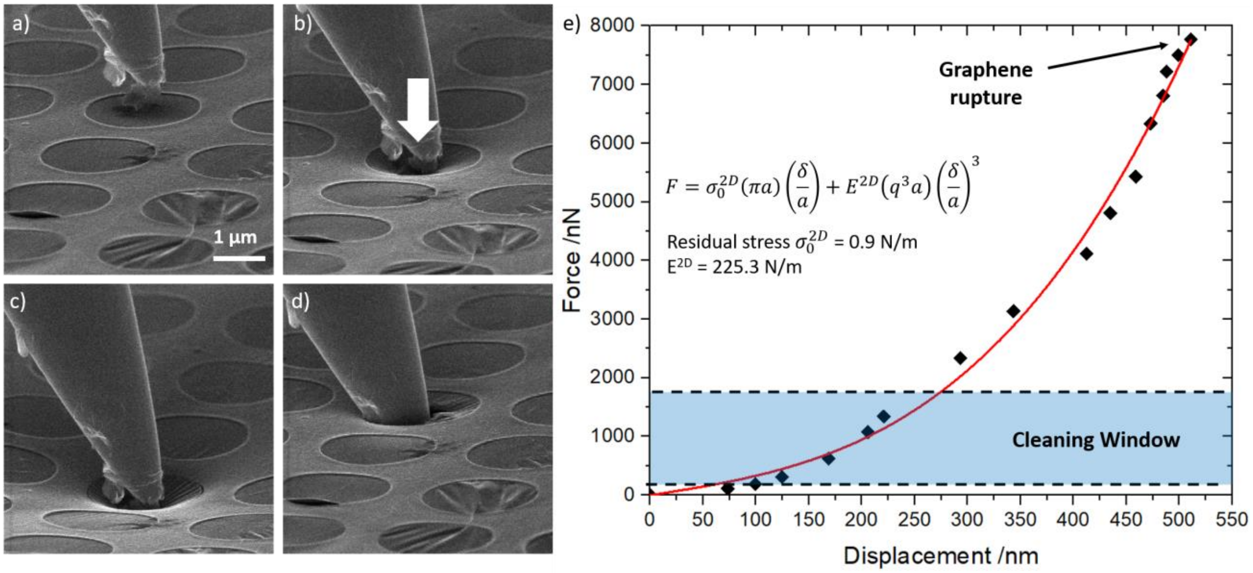

Supplementary Figure 3: Yield strength of graphene membranes. a)-d) Indentation of a free-standing graphene until rupture occurs. The membrane can sustain considerable deformation before rupture. e) Force displacement curve recorded during the experiment. The force was measured by tracking the deflection of the stiff copper support frame while the displacement was inferred from the difference between displacement at the tip and the edge of the hole in the support film. During cleaning the normal force stays well below the rupture point in all cases, even when more normal load than necessary was applied. By fitting the curve with a 2D elastic function (according to Lee et al. Science, 321, 5887 (2008)) the Young's modulus of graphene can be estimated as well as residual stresses. Here we obtain a 2D modulus of 225.3 N/m which translates to a Young's modulus of 672 GPa, assuming a layer thickness of 0.335 nm.

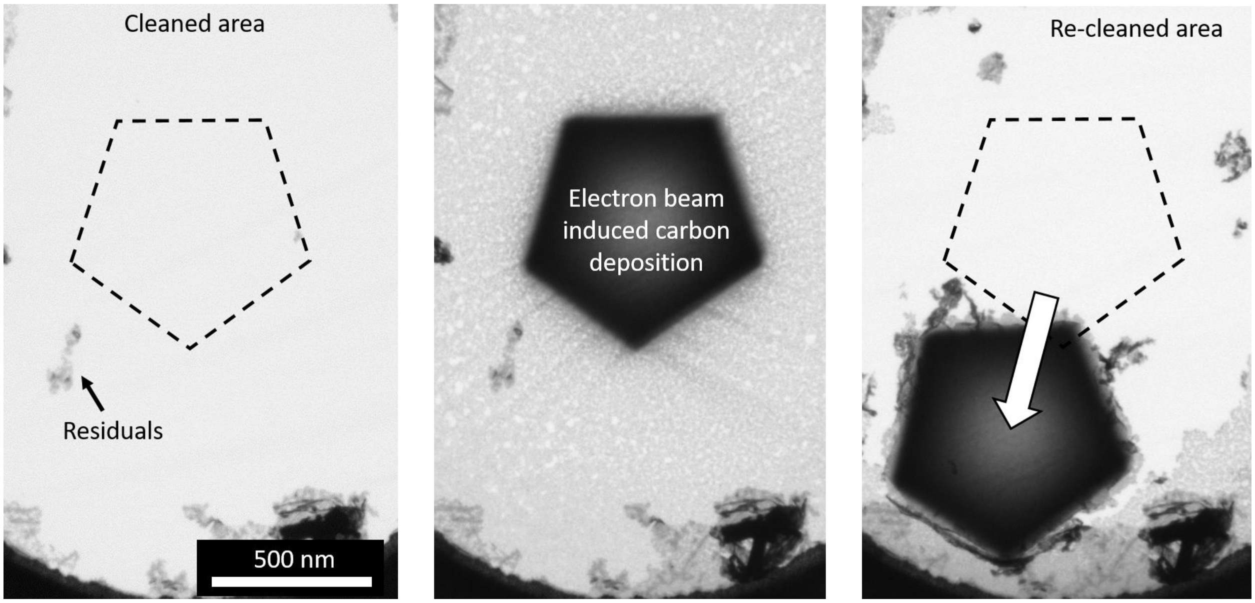

Supplementary Figure 4: Removal of purposely deposited carbon on a cleaned area. After cleaning of a graphene membrane, carbon is deposited in a pentagonal shape using a naphthalene gas-injection system. This carbon deposition can only be removed as a whole and pushed to the side of the membrane.

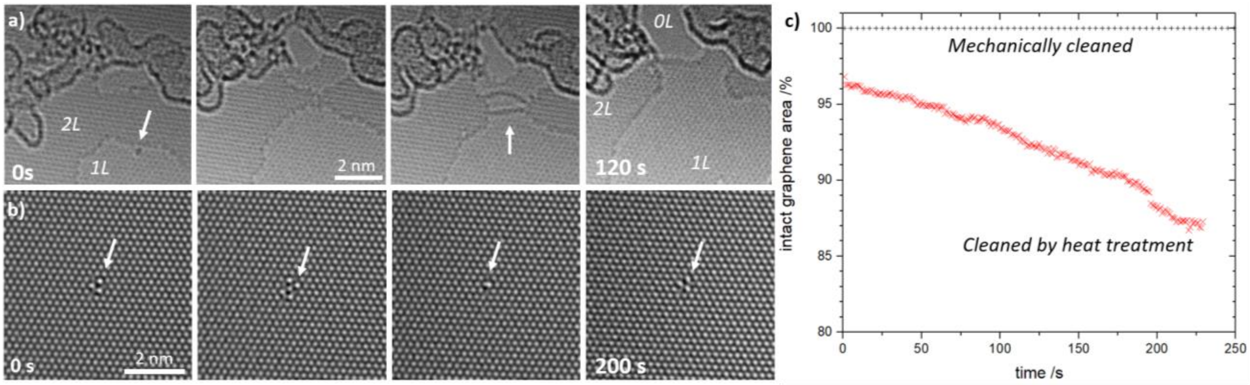

Supplementary Figure 5: Stability of graphene in TEM with conventional and mechanical cleaning. a) Dissolution of bilayer graphene after conventional cleaning by heat treatment. Mobile adatoms (arrow in the first image) enhance the dissolution process. On the contrary, mechanically cleaned graphene can be imaged for extended periods of time without degradation (b). Even in the presence of intrinsic defects, such as divacancies, no dissolution is found. c) Analysis of the intact area of graphene over time when viewed in TEM showing a continuous drop-of for conventionally cleaned samples.

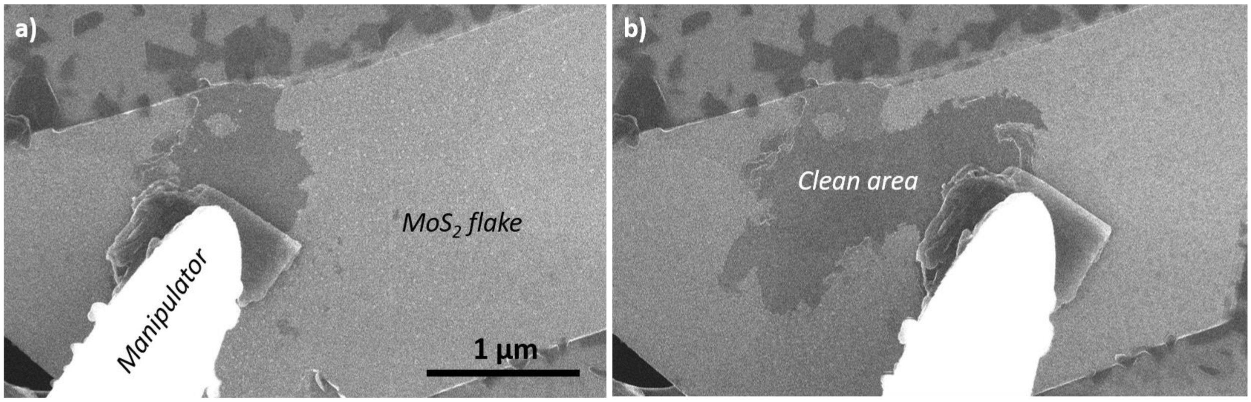

Supplementary Figure 6: *In situ* SEM cleaning of molybdenum disulfide. Mechanically exfoliated MoS2 is cleaned the same way as graphene (a & b), resulting in atomically clean areas on the surface.

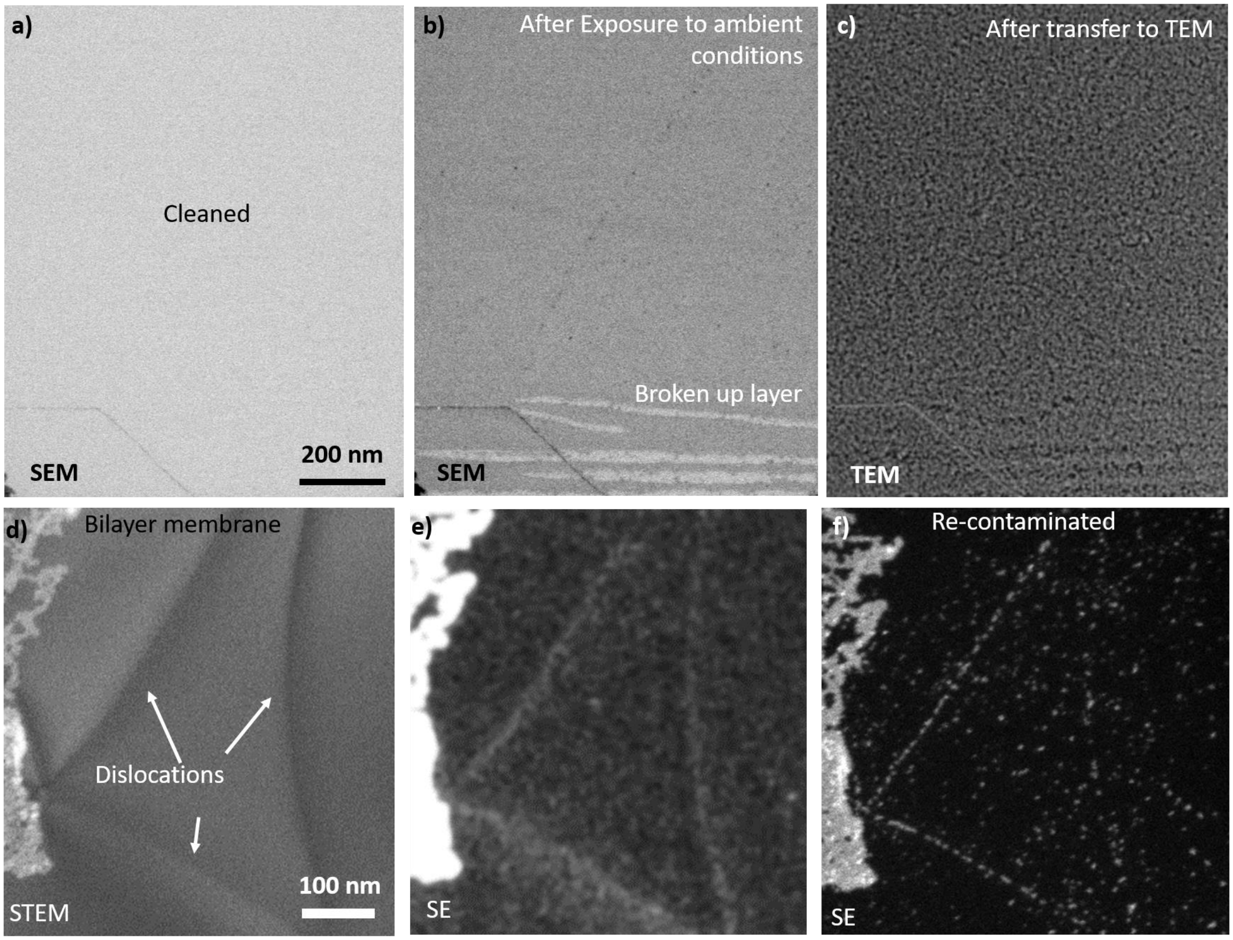

Supplementary Figure 7: Recontamination effects of cleaned membranes. a) SEM-STEM image of a cleaned membrane, showing a uniform contrast. After exposure to ambient conditions and re-insertion into the vacuum system of our SEM, a continuous layer of contamination is present (b). Only few cracks are present in the layer, which reveal a pristine surface. These cracks presumably appear due to folding. A second transfer and investigation of the contamination in TEM reveals a dense, but discontinuous layer of contamination, showing the effect of the vacuum system on the contamination layer (c). *In situ* contamination preferably grows at defects, such as dislocations. These defects are often present in bilayer membranes and can be seen in STEM (d) and surprisingly, also in the SE signal (e). During illumination with an electron beam, recontamination preferentially grows at those defects (f), which might be an effect of the surface topography or the increased emission of low-energy secondary electrons.

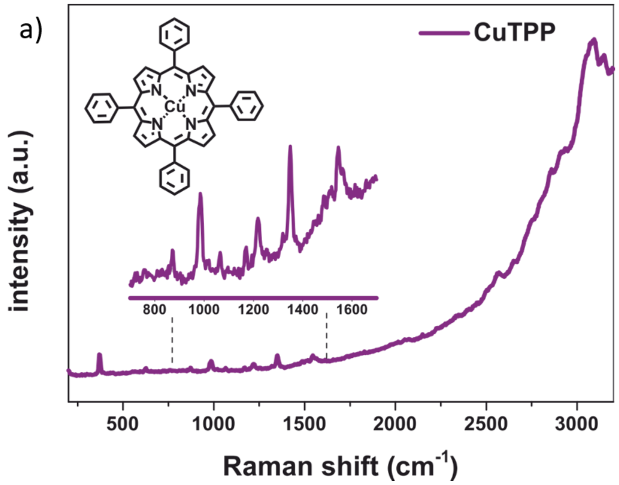

Supplementary Figure 8: Raman spectra of CuTTP supplied to cleaned surfaces. The spectra were measured at 532 nm. Spectrum of CuTPP alone (a) and of CuTPP on Graphene (b) showing a clear presence of the molecule after the application process.

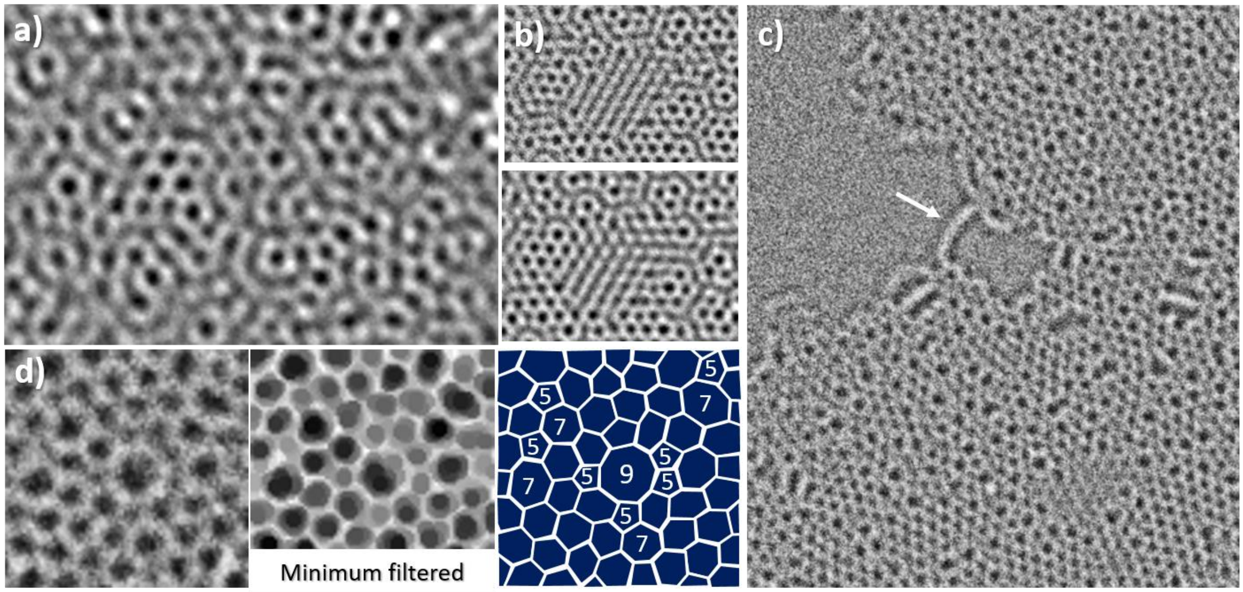

Supplementary Figure 9: Aspects of *in situ* growth of graphene layers. a) Triple layer showing an increasingly disordered structure. b) Bernal-type bilayer area, showing signs of in-plane dislocations (stripe-contrast) and AA-stacking (inverted lattice contrast). c) During the growth of the second layer, larger holes (arrow) are created, which are subsequently filled with additional carbon atoms. d) Formation of metastable 9-rings. Original image on the left, minimum filtered image in the middle and schematic drawing on the right.
